# Draft Genomes of Two Sordariomycete Fungi That Produce Novel Secondary Metabolites

**DOI:** 10.1128/genomeA.00291-15

**Published:** 2015-04-16

**Authors:** Blake W. Stamps, Lin Du, Carter A. Mitchell, Robert H. Cichecwicz, Bradley S. Stevenson

**Affiliations:** a Department of Microbiology and Plant Biology, University of Oklahoma, Norman, Oklahoma, USA; b Department of Chemistry and Biochemistry, University of Oklahoma, Norman, Oklahoma, USA; c Institute for Natural Products Applications and Research Technologies, University of Oklahoma, Norman, Oklahoma, USA

## Abstract

The genomes of two fungi isolated from soil (MEA-2) and sediment (SUP5-1) were sequenced. Both were members of the order *Hypocreales*, closely related to *Tolypocladium inflatum*, and capable of producing novel secondary metabolites. The draft genomes enabled the characterization of key biosynthetic pathways.

## GENOME ANNOUNCEMENT

S*ordariomycetes* are a diverse class of fungi, whose members are known to produce many important secondary metabolites, some with clinical importance (1–3). One member of the genus *Tolypocladium* was recently sequenced and shown to be capable of producing many different types of secondary metabolites (4). Two fungi were isolated in an effort to discover novel bioactive secondary metabolites, both of which belonged to the order *Hypocreales*, with close similarity to *Tolypocladium inflatum*. Given the association between the two isolates (MEA-2, also referred to as Salcha MEA-2 [5], and SUP5-1) and their close association with other metabolically talented fungi, their genomes were sequenced in an effort to determine their biosynthetic potentials.

Biomass from each isolate was extracted using the MoBio Power Biofilm DNA extraction kit to produce enough DNA for genomic sequencing. Using this DNA, the genome of MEA-2 was sequenced using two MiSeq runs at 2 × 150 and 2 × 250 bp. The genome of SUP5-1 was also sequenced on the Illumina HiSeq 2000 system with a 2 × 100-bp rapid run. Both batches of sequence data were filtered for quality and then assembled in CLC Genomics Workbench version 7.0 (Qiagen Inc.). Genomic DNA was also sequenced from each isolate using two Pacific Biosciences RS II SMRT Cells with the most recent P6-C4 sequencing chemistry to produce a final assembly. The Pacific Biosciences sequence data were assembled using the SMRT Portal bioinformatics software suite.

After assembly, MEA-2 contained 44 contigs >500 bp, and SUP5-1 contained 400 contigs. The *N*_50_ lengths were 1.148 Mbp for MEA-2 and 168 kbp for SUP5-1, the largest contigs of each being 2.615 Mbp and 804 kbp, respectively. The Illumina sequencing reads were mapped to the final long-read assembly using CLC Genomics Workbench version 7.0 (Qiagen Inc.), to ensure that the final assembly produced was of high consensus to a previous Illumina assembly.

The full-length 18S rRNA gene sequence for each isolate was assembled using EMIRGE (6) to confirm the taxonomic positions of MEA-2 and SUP5-1. Both fungi are likely different species of the genus *Tolypocladium* and closely related to *Tolypocladium inflatum*. The assembled genomes were used to assess the biosynthetic potential of both isolates using the Antibiotics and Secondary Metabolite Analysis Shell (antiSMASH) (7, 8). Both fungi contained a large number of secondary metabolite synthesis clusters, with SUP5-1 potentially containing more biosynthetic clusters than MEA-2. The biosynthetic pathways of both organisms cover a wide range of putative metabolite families, including polyketide, nonribosomal peptide, terpene, and hybrid compounds.

The genomes and their analysis described here have provided important insights to the biosynthetic potential of these two members of the *Hypocreales*. These isolates and other members of the *Hypocreales* will be the focus of further efforts to discover novel, bioactive secondary metabolites.

### Nucleotide sequence accession numbers. 

This whole-genome shotgun sequencing project has been deposited at GenBank under the accession numbers JPIJ00000000 for MEA-2 and JPHH00000000 for SUP5-1. Raw reads were deposited to the NCBI SRA under the accession numbers SRX864103 and SRX864916.
